# Solid base catalysts derived from Ca–Al–X (X = F^−^, Cl^−^ and Br^−^) layered double hydroxides for methanolysis of propylene carbonate†

**DOI:** 10.1039/c7ra10832j

**Published:** 2018-01-02

**Authors:** Yunhui Liao, Feng Li, Yanfeng Pu, Feng Wang, Xin Dai, Ning Zhao, Fukui Xiao

**Affiliations:** State Key Laboratory of Coal Conversion, Institute of Coal Chemistry, Chinese Academy of Sciences 27# South Taoyuan Road Taiyuan 030001 P. R. China lifeng2729@sxicc.ac.cn xiaofk@sxicc.ac.cn; University of Chinese Academy of Sciences Beijing 100049 P. R. China

## Abstract

The Ca–Al and Ca–Al–X (X = F^−^, Cl^−^ and Br^−^) catalysts were prepared *via* thermal decomposition of Ca–Al layered double hydroxides (LDHs), and tested for methanolysis of propylene carbonate (PC) to produce dimethyl carbonate (DMC). The catalytic performance of these catalysts increased in the order of Ca–Al–Br^−^ < Ca–Al < Ca–Al–Cl^−^ < Ca–Al–F^−^, which was consistent with the strong basicity of these materials. The recyclability test results showed that the addition of Al and halogens (F^−^, Cl^−^ and Br^−^) not only stabilized the CaO but also improved the recyclability of the catalysts. Particularly, the Ca–Al–F^−^ catalyst exerted the highest stability after 10 recycles. These catalysts have an important value for the exploitation of DMC synthesis by transesterification of PC with methanol.

## Abstract 2

The CA-F^−^ catalyst modified with Al^3+^ and F^−^ was highly active and recyclable for dimethyl carbonate synthesis.

## Graphical abstract

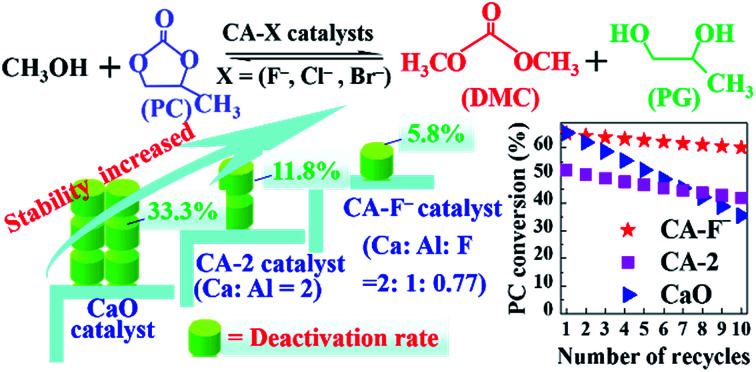

## 1 Introduction

Dimethyl carbonate (DMC), as a green organic fine chemical intermediate, is widely used in carbonylation, methylation and methoxylation reactions.^1,2^ Several ways are developed for DMC synthesis, such as oxidative carbonylation of methanol,^3–5^ transesterification of methanol with cyclic carbonate,^6–9^ alcoholysis of urea^10–12^ and direct synthesis from carbon dioxide and methanol.^13–16^ As compared to other routes, DMC synthesis by transesterification of propylene carbonate (PC) with methanol under mild conditions is a promising and environmentally benign route, which has earned much attention of researchers.^17,18^

Several catalysts, such as alkali metals,^19^ KF supported catalysts,^20^ zeolites,^21^ ion exchange resins,^22,23^ double metal cyanides,^17^ hydrotalcites,^24,25^ metal oxides and mixed metal oxides^26–29^ have been investigated for the transesterification of PC with methanol. However, the high reaction temperature (403–443 K) restricts the DMC yield due to the equilibrium limitation.^30^ Therefore, efficient catalysts with high activity at low temperature are highly demanded. It was reported that CaO was effective for the reaction at room temperature,^31^ but the recyclability was poor due to the leaching of the active phase in the reaction medium which limited its industrial application.^32,33^ In order to improve the recyclability of the CaO for the synthesis of glycerol carbonate from glycerol and dimethyl carbonate, Lu *et al.*^34^ added the Al into the CaO by an extrusion method, which effectively improved the stability of CaO. Kocík *et al.*^35^ prepared the Ca–Al oxide catalysts by coprecipitation method for the biodiesel production, and found that the obtained catalysts was stable with nearly no calcium leaching.

In addition, it is well known that the basicity of the catalysts plays an important role in the transesterification of PC with methanol.^20^ It is possible to modulate the basicity of the catalysts by modification of halogen anions. Dai *et al.*^36^ employed the BaX_2_ (X = F^−^, Cl^−^ and Br^−^)-promoted Y_2_O_3_ catalysts for the oxidative dehydrogenation of ethane, and the CO_2_-TPD results indicated that the halogen anions effectively improved the basicity of the catalysts, thereby improved the conversion rate of the catalytic reaction. Au *et al.*^37^ reported the F^−^ and Br^−^ modified La_2_O_3_ catalyst for the oxidative coupling of methane, and the CO_2_ adsorption characterization also demonstrated that introduction of F^−^ and Br^−^ increased the basicity of the catalysts. Wu *et al.*^38^ discovered that the fluorine-modified Mg–Al mixed oxides as a solid base with variable basic sites and tunable basicity were effective for the synthesis of propylene glycol methyl ether (PM) from methanol and propylene oxide (PO). Gao *et al.*^39^ synthesized a series of fluorine-containing Cu/Zn/Al/Zr hydrotalcite-like compound catalysts which were tested for CO_2_ hydrogenation to methanol. It was found that introduction of F^−^ enhanced the surface basicity, and significantly increased the selectivity of methanol.

Layered double hydroxides (LDHs) can be represented by the formula of [M_1−*x*_^2+^M_*x*_^3+^(OH)_2_]^*x*+^[(A^*n*−^)_*x*/*n*_]^*x*−^·*m*H_2_O, where M^2+^ and M^3+^ are divalent and trivalent metal, respectively; the value of *x* is equal to the molar ratio of M^3+^/(M^2+^ + M^3+^) and A^*n*−^ is the anion which has the exchangeable capacity in compensating charge position.^40,41^ After calcination, the obtained mixed oxides possess enough Lewis base sites and uniform distribution.^42^ With the intercalation of different cations and anions, the basicity of the catalysts can be modulated.

In this work, a series of Ca–Al mixed oxide catalysts were synthetized by thermal decomposition of Ca–Al–X (X = F^−^, Cl^−^ and Br^−^) layered double hydroxides (LDHs), and were tested for the transesterification of PC with methanol. The effect of Al on the stabilization of the active CaO component and the influence of the halogen anions (F^−^, Cl^−^ and Br^−^) on the basicity of the Ca–Al mixed oxide catalysts were investigated. This work has meaningful and practical applicative values for the stabilization and basicity regulation of CaO catalyst, and provides a new idea for the stabilization of the catalyst applied to the transesterification.

## 2 Experimental

### 2.1 Catalysts preparation

The Ca–Al LDH with Ca/Al molar ratio of 2 was prepared by coprecipitation method. Typically, appropriate amounts of CaCl_2_ and AlCl_3_·6H_2_O (the molar ratio of Ca/Al was 2) were dissolved in 100 mL deionized decarbonated water to prepare solution A, while appropriate amounts of NaOH were dissolved in 100 mL deionized decarbonated water to prepare solution B. Subsequently, both solutions were added dropwise by peristaltic pump to 250 mL water–ethanol (2 :  3, v/v) solution at 333 K under vigorous stirring in inert atmosphere (N_2_), and the pH was kept at 10. The suspended liquid was aged at 333 K for 24 h. After filtration, the precipitate was washed with deionized decarbonated water until the pH of the filtrate was near 7. Then, the filtration cake was dried at 353 K in vacuum for 24 h. Finally, the catalyst was obtained by calcination in N_2_ atmosphere at 1073 K for 6 h. The precursor and the calcined catalyst were labelled as CAP-2 and CA-2, respectively.

The Ca–Al–X (X = F^−^, Cl^−^ and Br^−^) LDHs were prepared by the coprecipitation method. Typically, appropriate amounts of calcium and aluminum chloride salts (the molar ratio of Ca/Al was 2) were dissolved in a 100 mL deionized decarbonated water (solution C). Then, appropriate amounts of NaOH and Na–X (X = F^−^, Cl^−^ and Br^−^) were dissolved in a 100 mL deionized decarbonated water (solution D). The molar ratio of Ca/X was 2.5. The following steps were the same as the preparation methods of CAP-2 and CA-2. The precursors and calcined catalysts were labelled as CAP-X and CA-X (X = F^−^, Cl^−^ and Br^−^), respectively.

For comparison, the CaO catalyst was prepared by the coprecipitation method. Typically, appropriate amounts of calcium chloride salts were dissolved in a 100 mL deionized decarbonated water (solution E). Then, appropriate amounts of NaOH were dissolved in a 100 mL deionized decarbonated water (solution F). The following steps were the same as the preparation methods of CA-2.

### 2.2 Catalysts characterization

The X-ray powder diffraction (XRD) was performed on a Bruker D8 Advance (Germany) diffractometer, using Cu Kα radiation at 40 kV and 50 mA. The scan rate was 3° min^−1^ in the 2*θ* range from 5° to 80°.

The surface morphologies of the catalysts were collected with a JSM-7001F scanning electron microscope.

The Fourier transform infrared (FT-IR) spectra were acquired using a Nicolet Nexus 470 FT-IR spectrometer range from 400 to 4000 cm^−1^ with a 4 cm^−1^ resolution.

Thermogravimetric (TG) analyses were measured with Rigaku TG 8120 equipment. The samples were heated at a rate of 10 K min^−1^ from room temperature to 1173 K in N_2_ atmosphere.

The N_2_ adsorption–desorption isotherms measurement at 77 K employed the Brunauer–Emmett–Teller method. The specific surface areas of catalysts were determined from the nitrogen adsorption isotherms.

The metal (Ca and Al) elemental chemical analysis was measured with the inductively coupled plasma-optical (ICP) emission spectroscopy (Thermo iCAP 6300). Halogen anions were measured with an ionic chromatography (881 Compact IC Pro, Metrohm) with conductivity detection.

X-ray photoelectron spectroscopy (XPS) was analyzed by Axis Ultra DLD spectrometer with a monochromatic Al Kα (1486.8 eV) source under ultrahigh vacuum. The binding energies were calibrated internally using the C 1s peak with *E*_b_ = 284.8 eV. The experimental error was within ±0.1 eV.

CO_2_ temperature programmed desorption (CO_2_-TPD) was performed on Builder PCA-1200 chemical adsorption instrument with a thermal conductivity detector using He as carrier gas. The catalyst was placed in a quartz U-tube reactor and pre-treated under helium flow at 1073 K for 1 h, and then cooled to room temperature under helium flow. The adsorption process was performed with CO_2_ (30 mL min^−1^) at room temperature for 30 minutes, and desorption process was measured from room temperature to 1173 K at a heating rate of 10 K min^−1^ under helium flow. The thermal conductivity detector (TCD) was operated in differential mode and the signal transferred to a data acquisition computer.

### 2.3 Catalytic reaction

**Scheme 1:**
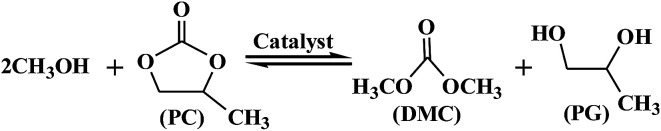
Reaction scheme for the synthesis of DMC.

The DMC synthesis from transesterification of methanol with PC can be depicted in Scheme 1. All catalysts were pre-treated at 1073 K in N_2_ atmosphere for 1 h before reaction. The transesterification was carried out in a three neck flask with Dimroth condenser containing designed amount (mmol) of reactants and catalysts. The products were analyzed by gas chromatography (GC-950) equipped with TCD detector. The separation column was made of *Φ* 3 mm × 3 m stainless steel column filled with GDX-203 stationary phase. The conversion of PC and selectivity of DMC were calculated by the following equations:1
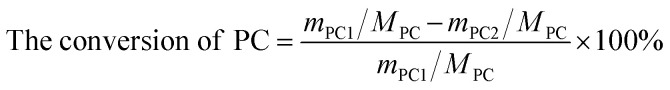
2
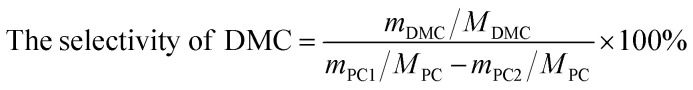
*m*_DMC_ is the mass of formed DMC, *M*_DMC_ is the molecular weight of DMC, *m*_PC1_ is the mass of PC in the feeds, *m*_PC2_ is the mass of PC in the products, and *M*_PC_ is the molecular weight of PC.

## 3 Results and discussion

### 3.1 Characterization of the catalysts

**Fig. 1:**
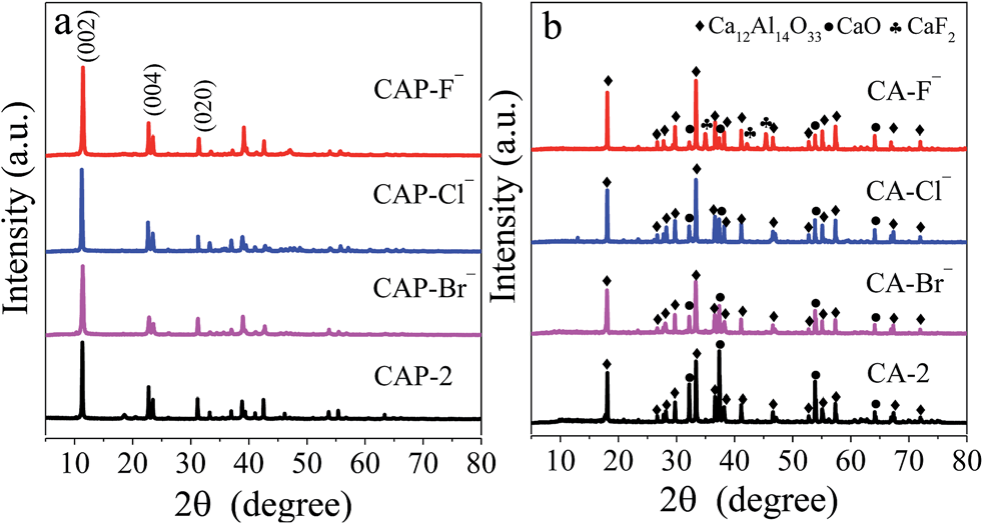
XRD patterns of (a) CAP-2, CAP-X and (b) CA-2, CA-X samples.

The XRD technique is employed to characterize the structure of LDH precursors, and the patterns of the CAP-2 and CAP-X samples are shown in Fig. 1a, in which diffraction peaks correspond to (002), (004), and (020) planes of the Ca–Al LDHs are observed, suggesting the well-developed layered structure.^43^ The TG-DTG curves of the samples are shown in Fig. S1.† The thermal stability of LDH precursors are in the order of CAP-F^−^ > CAP-Cl^−^ > CAP-2 > CAP-Br^−^.

After calcination at 1073 K, the phases of the LDH disappear, while cubic phase of CaO and mayenite are observed as shown in Fig. 1b. Specially, the CaF_2_ is detected for the CA-F^−^ sample.

**Fig. 2:**
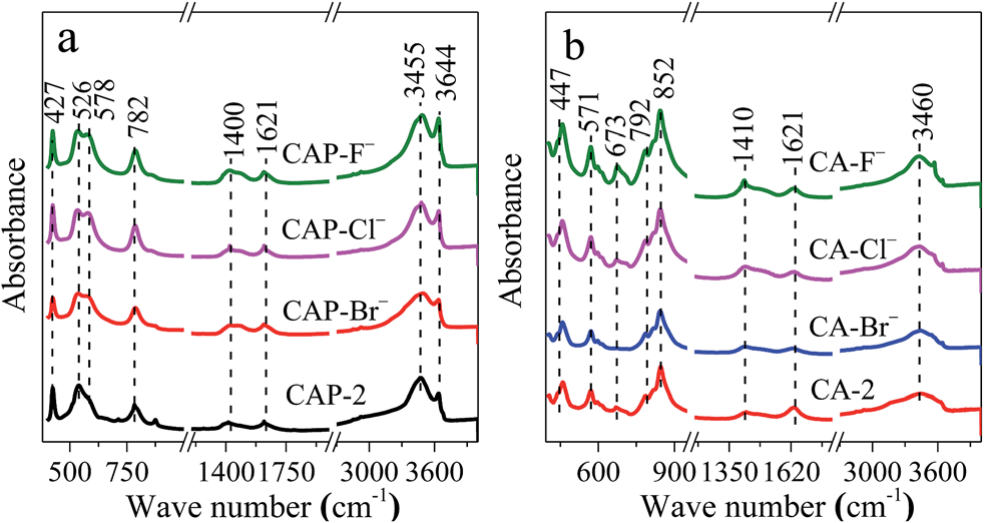
Infrared spectra of (a) CAP-2, CAP-X and (b) CA-2, CA-X samples.

The infrared spectra are employed to study the structural features of the samples. For all CAP-X samples (Fig. 2a), the absorption band at 3644 cm^−1^ due to the O–H stretching vibration of free water is observed.^10^ The broad band at 3455 cm^−1^ can be ascribed to the stretching mode of hydroxyl groups, both from the layers and interlayer water molecules.^44^ Moreover, the band at 1621 cm^−1^ attributed to the bending deformation of molecular water is presented.^45^ The band attributed to the asymmetric stretching of C–O bonds in carbonate ions is also observed at around 1400 cm^−1^.^46,47^ In fact, it is inevitable to suffer some carbonates due to high alkaline surface of the samples.^48,49^ In addition, the vibrations at around 782, 578, 526 and 427 cm^−1^ are assigned to the characteristic vibration of M–O and M–OH (M = Ca and Al).^48^ It is noteworthy that the shape of the absorption band centered at around 578 and 526 cm^−1^ for CAP-2 sample is asymmetric, while the halogen anions modified CAP-X samples are relatively symmetric, which suggests that the vibration of M–O and M–OH is modified owing to the anionic group in the layered structure of LDH.

After calcination, great changes are found for the CA-X samples (Fig. 2b) *e.g.* the intensity of the band at 3460 and 1621 cm^−1^ assigned to the vibration band of hydroxyl groups decrease, suggesting that the water in the interlayer is removed.^10^ The bands at around 447, 571, 673, 792 and 852 cm^−1^ ascribed to the characteristic vibration of Ca–O and Al–O shift to the higher wave number due to the phase transformations.^48^ The spectrum is dominated by a strong band at 852 cm^−1^ and the intensity of this band increases in the order of CA-F^−^ > CA-Cl^−^ > CA-2 > CA-Br^−^. For the CA-F^−^ sample, the highest intensity of this absorption band indicate the change in the environment of M–O (M = Ca and Al) systems due to the introduction of F^−^ which may increase the stability of the M–O group.

The textural parameters and compositions of the catalysts are summarized in Table 1. Compared to the CA-2 catalyst, the specific surface areas and pore volume of the catalysts increase with introduction of F^−^ and Cl^−^. The CAP-F^−^ and CAP-Cl^−^ LDH precursors have more united structures, which result in more amounts of small and uniform particle size after thermal treatment as supported by the SEM analysis (Fig. S2†), and thus lead to the increased specific surface areas and pore volume. ICP results show that the bulk metal compositions of the catalysts are similar to that in the mother liquor, indicating that the metal ions precipitated completely in the preparation process. While XPS results indicate that the Ca is enriched on the surface which is more remarkable for the halogen anions modified catalysts. Thus, it can be concluded that the surface compositions of the catalysts can be modulated by modification of the halogen anions. Specially, the F^−^ shows the most significant impact on the surface Ca : Al atomic ratio.

**Table 1:** Textural parameters and compositions of the catalysts^a^

Catalysts	*S*_BET_ (m^2^ g^−1^)	*V*_pore_ (cm^3^ g^−1^)	Compositions^b^ (mol%)			
			Ca	Al	X	Ca : Al
CA-F^−^	45.0	0.38	52.9 (54.8)	26.6 (25.7)	20.5 (19.5)	1.99 (2.13)
CA-Cl^−^	40.3	0.33	53.0 (54.1)	26.4 (26.1)	20.6 (19.8)	2.01 (2.07)
CA-Br^−^	32.7	0.22	52.8 (53.7)	26.6 (26.4)	20.6 (19.9)	1.98 (2.03)
CA-2	37.6	0.26	66.6 (66.9)	33.4 (33.1)	0	1.99 (2.02)

a X = F^−^, Cl^−^ and Br^−^.

b The values outside and inside the parentheses were obtained by ICP or ionic chromatography and XPS measurements, respectively.

**Fig. 3:**
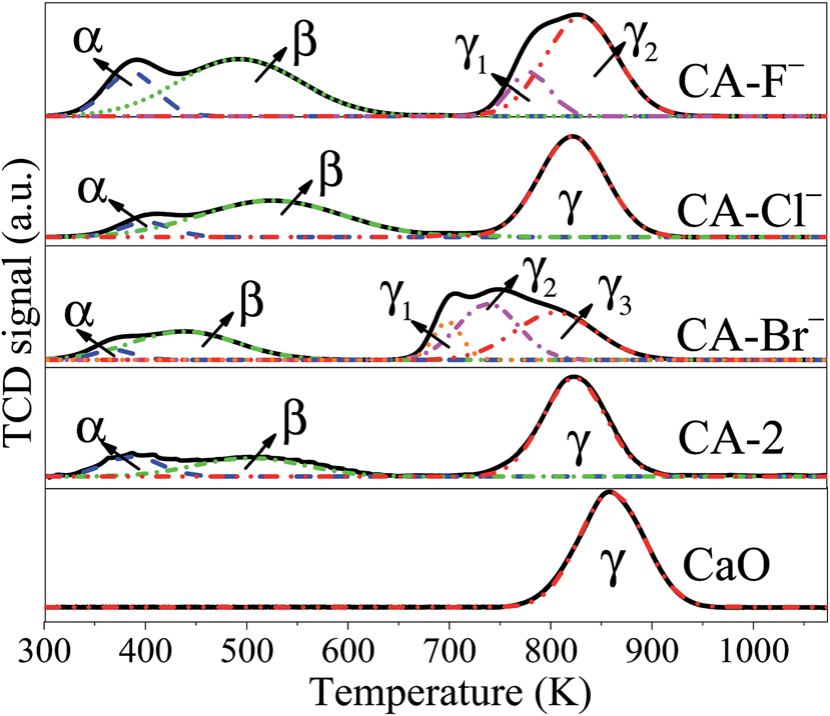
CO_2_-TPD profiles of the catalysts.

The CO_2_-TPD profiles are shown in Fig. 3. For the pure CaO catalysts, only one desorption peak ascribed to the strong basic sites is observed. Obviously, with the addition of Al and halogen anions, three broad desorption peaks for each catalyst are observed, which indicate the different adsorption sites for CO_2_. For the CA-2 catalyst, the weak (α peak), moderate (β peak) and strong (γ peak) basic sites can be ascribed to basic OH^−^ group, Al^3+^–O^2−^ and Ca^2+^–O^2−^ pairs and unsaturated O^2−^ anions, respectively.^49,50^ While the CA-X catalysts are consisted of more complex basic groups *e.g.* the basic OH^−^ group can result in the weak basic site, the Al^3+^–X^−^, Ca^2+^–X^−^, Al^3+^–O^2−^ and Ca^2+^–O^2−^ pairs can lead to the moderate basic site, the coordinatively unsaturated X^−^ and O^2−^ anions can result in the strong basic site.^38,51,52^ The CO_2_ uptakes on each catalyst calculated from the CO_2_-TPD peaks area are listed in Table 2. The amounts of the strong and total basic site of the catalysts increase in the same order of CA-F^−^ > CA-Cl^−^ > CA-2 > CA-Br^−^. Since the ionic radii of halogen anions increase in the order of F^−^ < Cl^−^ < Br^−^, the superficial F^−^ is more effective to substitute O^2−^ than Cl^−^ and Br^−^, which may accounts for the highest basicity of the CA-F^−^ catalyst.^53^ Besides, the CO_2_ desorption peak ascribed to the strong basic site of CA-F^−^ catalyst shifts to higher temperature, suggesting that the strong basicity of the catalyst increase with modification of F^−^. It can be interpreted as the generation of coordinatively F^−^ and O^2−^ unsaturated anions.^38,51,52^

**Table 2:** The CO_2_-TPD results of the catalysts

Catalysts	CO_2_ uptakes (mmol g^−1^)			Total basicity (mmol g^−1^)
	α	β	γ	
CA-F^−^	0.11	0.34	0.56	1.01
CA-Cl^−^	0.05	0.33	0.49	0.87
CA-Br^−^	0.06	0.23	0.36	0.65
CA-2	0.10	0.26	0.44	0.80
CaO	0	0	0.60	0.60

### 3.2 Catalytic performance and recyclability of the catalysts

**Fig. 4:**
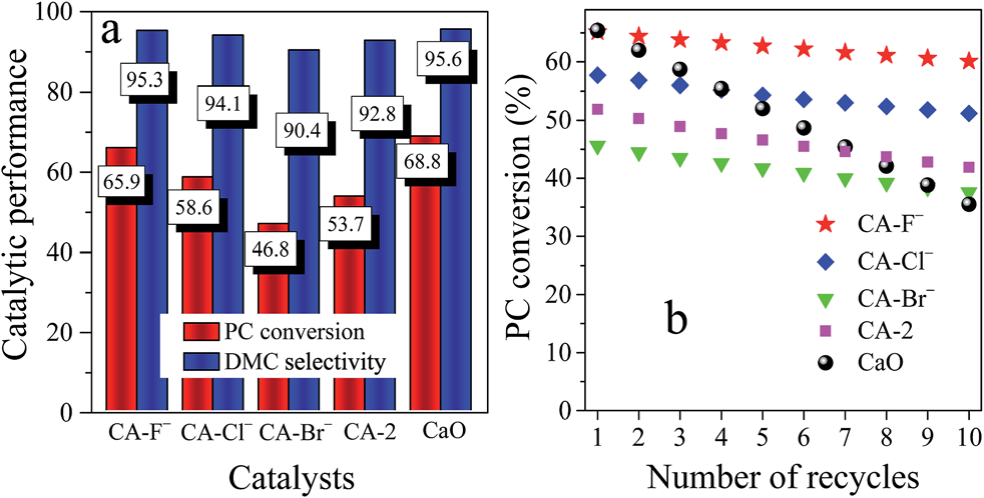
The catalytic performance and recyclability of the CA-X, CA-2 and CaO catalysts. Reaction conditions: *n*(methanol)/*n*(PC) = 12, catalyst weight = 2 wt% of total reactants, *T* = 333 K, *t* = 2 h.

The effect of reaction parameters on catalytic performance of the catalysts was investigated (Fig. S3†) and the catalytic results under the optimized condition were shown in Fig. 4. The pure CaO catalyst possessed good catalytic performance with the PC conversion of 68.8% and the DMC selectivity of 95.6% (Fig. 4a). However, the PC conversion decreased to 35.5% after 10 recycles (Fig. 4b), suggesting the poor recyclability which is consistent with the previously reported literature.^32,33^ Upon addition of Al into the CaO, although the PC conversion of the CA-2 catalyst was reduced to 53.7%, the decrement of PC conversion after 10 recycles was only 11.8%, which suggest that the Al effectively improved the stability of CaO. Furthermore, the catalytic performance and recyclability of the catalysts both increased with modification of F^−^ and Cl^−^. The CA-F^−^ catalyst possessed the high catalytic performance with the PC conversion of 65.9% and DMC selectivity of 95.3%, which was similar to the CaO catalyst. Moreover, the catalyst still possessed a higher PC conversion (60.1%) after 10 recycles, and the decrement of PC conversion was only 5.8%. While for the CA-Br^−^ catalyst, the PC conversion was lower than the CA-2 catalyst in spite of the increased stability.

During the reaction, the catalyst modified by the F^−^ exerted the high catalytic performance. Previous reports described that the active K^+^ and F^−^ presented on the surface of KF/Al_2_O_3_ catalyst were responsible for the transesterification of PC with methanol.^20,54,55^ Zhu *et al.*^56^ demonstrated that the basic Al–[OH⋯F]^−^ species was effective for the activation of methanol. Ando *et al.*^57^ and Chen *et al.*^58^ also emphasized the significant effect of coordinately unsaturated F^−^ as the basic sites, which effectively activated the methanol. According to the results of CO_2_-TPD, the introduction of F^−^ greatly increased the amounts of the basic sites, and thus significantly increased the catalytic performance of the catalysts. The catalysts with high basicity lowered the free energy of the transesterification, which accelerated the generation and desorption of the products from the active basic sites of the catalysts, and thus increased the PC conversion.^59^

**Fig. 5:**
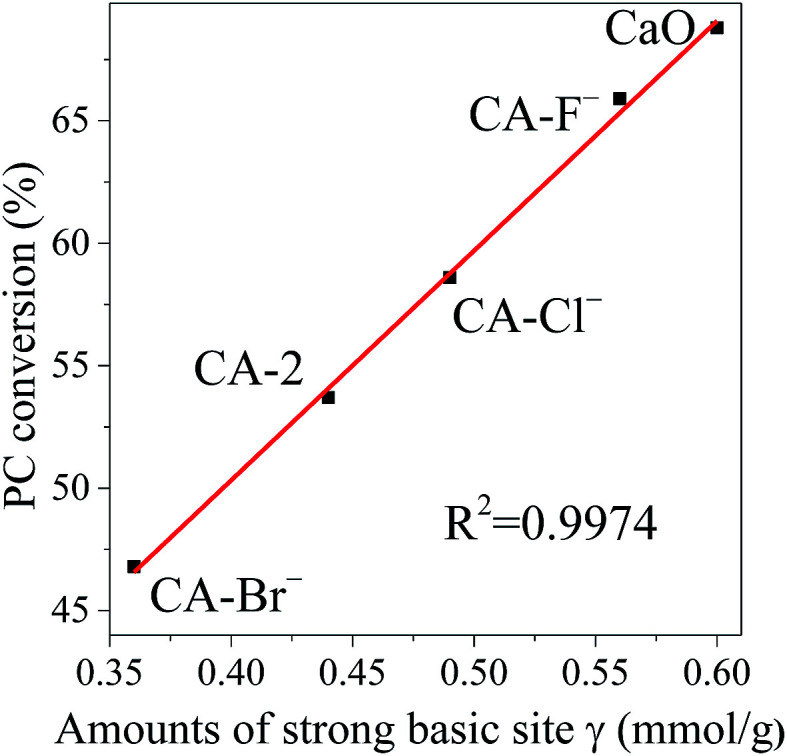
The relationship between PC conversion and amounts of the strong basic site γ. Reaction conditions: catalyst weight = 2 wt% of total reactants, *n*(methanol)/*n*(PC) = 12, *T* = 333 K, *t* = 2 h.

Associated with the CO_2_-TPD characterization results, the catalysts are consisted of different basic sites. Therefore, the relationship between PC conversion and different basic sites of the catalyst is investigated. It is noticeable that the PC conversion is linearly related with the amounts of the strong basic sites γ as shown in Fig. 5. It indicates that although the transesterification of PC with methanol occurred over various basic sites, the strong basic site γ plays main role on the reaction.

### 3.3 A discussion of the reaction mechanism

**Scheme 2:**
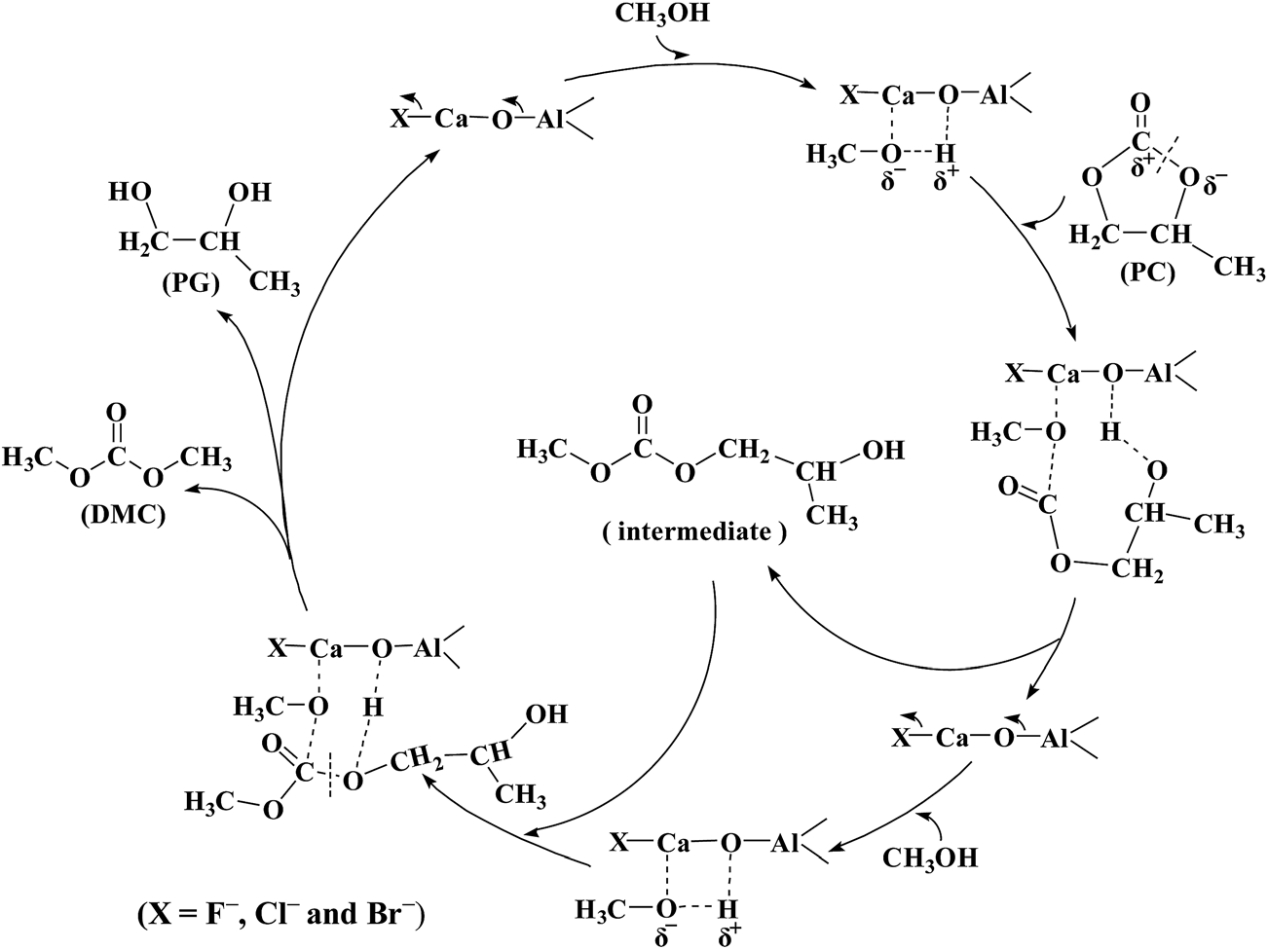
Possible reaction mechanism of DMC synthesis.

Based on previous researches^18,26,60^ and the results in this work, a possible reaction mechanism of DMC synthesis from PC with methanol is proposed and depicted in Scheme 2. The surface basic sites of catalysts activated the methanol, which cleaved PC to form 2-methyl-hydroxyethyl methyl carbonate as an intermediate. The intermediate further reacted with the activated methanol to produce DMC and PG as product. For this reaction, the main role of solid base catalyst is to activate CH_3_OH through the abstraction of H^*δ*+^ by basic site. It is possible that the higher the basicity of the catalyst, the more negative is the charge of CH_3_O^*δ*−^, and thus easily promoted the reaction activity of DMC synthesis.

### 3.4 Stability of the catalysts

**Fig. 6:**
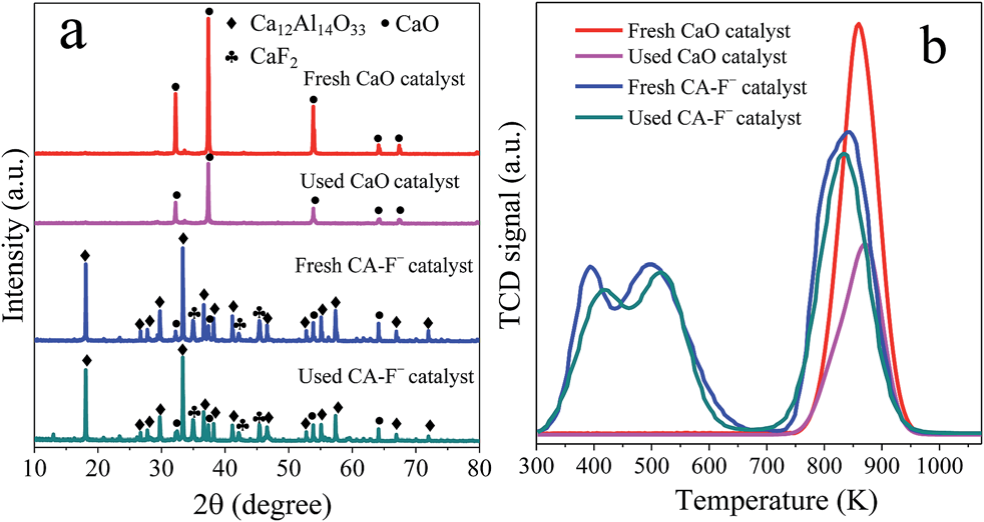
(a) XRD patterns and (b) CO_2_-TPD profiles of the fresh and used CaO and CA-F^−^ catalysts.

Fig. 6 shows the XRD patterns and CO_2_-TPD profiles of the fresh and used CaO and CA-F^−^ catalysts. For the pure CaO catalyst, the diffraction peaks at 2*θ* of 32.2°, 37.4°, 53.8°, 64.2° and 67.4° corresponded to the phase of CaO were weakened after 10 recycles. Combined with the CO_2_-TPD profiles (Fig. 6b), the adsorption capacity of the CO_2_ on the surface of the CaO catalyst significantly decreased after 10 recycles (Fig. 6a).^59,61^ Therefore, the dramatically decreased catalytic activity of the CaO catalyst could be ascribed to the decreased amounts of the surface basic sites. For the CA-F^−^ catalyst, the intensity of CaO phase and the CO_2_ adsorption capacity did not decrease significantly. Moreover, the elemental analysis (Table 3) shows that the content of F and Ca remain almost unchanged after 10 recycles, suggesting the high stability of the CA-F^−^ catalyst.

**Table 3:** The bulk compositions of the fresh and used catalysts

Catalysts	Bulk compositions (mol%)		
	Ca^a^	Al^a^	F^b^
Fresh CA-F^−^ catalyst	52.9	26.6	20.5
Used CA-F^−^ catalyst	51.5	28.3	20.2

a Determined by the ICP.

b Determined by the ionic chromatography.

## 4 Conclusions

In this work, a series of Ca–Al solid base catalysts were prepared *via* thermal decomposition of Ca–Al LDHs, and were effectively for the transesterification of PC with methanol. The characterization results demonstrated that Al and different halogen anions (F^−^, Cl^−^ and Br^−^) controlled the structural and chemical properties of the catalysts and affected the catalytic performance for the transesterification reaction. The PC conversion was linear to the strong basic amounts of the catalysts. The CA-F^−^ catalyst possessed the highest surface basicity and exerted the highest catalytic performance. The highest PC conversion of 65.9% and DMC selectivity of 95.3% had been reached with methanol : PC molar ratio of 12, catalysts weight of 2 wt% total reactants, and reaction time of 2 h. The addition of Al and halogen anions (F^−^, Cl^−^ and Br^−^) effectively improved the recyclability of catalysts. The CA-F^−^ catalyst maintains the highest activity after 10 recycles and the decrement of PC conversion was only 5.8%. The unchanged XRD pattern and chemical compositions of the use CA-F^−^ catalyst after 10 recycles showed the high recyclability and stability. The work reported herein is expected to advance the preparation method of high-efficiency solid base catalysts used in the transesterification of propylene carbonate with methanol, which has good prospects for industrial application with many advantages such as easy catalyst separation and better recyclability.

## Conflicts of interest

There are no conflicts to declare.

## Supplementary Material

RA-008-C7RA10832J-s001
