# Supplementary material for: Solid base catalysts derived from Ca–Al–X (X = F−, Cl− and Br−) layered double hydroxides for methanolysis of propylene carbonate
Source: RSC Adv. 2018 Jan 2;8(2):785–91. doi: 10.1039/c7ra10832j (PMC9076883; doi:10.1039/c7ra10832j)
Supplement: RA-008-C7RA10832J-s001 [file RA-008-C7RA10832J-s001.pdf]

Electronic Supplementary Material (ESI) for RSC Advances.
This journal is © The Royal Society of Chemistry 2017

Supplementary information

Solid base catalysts derived from Ca-Al-X (X = F^-, Cl^- and Br^-) layered double hydroxides for methanolysis of propylene carbonate

Yunhui Liao,[ab] Yanfeng Pu,[a] Feng Wang,[a] Feng Li,[*a] Xin Dai,[ab] Ning Zhao[a] and Fukui Xiao[*a]

[a] State Key Laboratory of Coal Conversion, Institute of Coal Chemistry, Chinese Academy of Sciences, Taiyuan 030001, P. R. China

[b] University of Chinese Academy of Sciences, Beijing 100049, P. R. China

*Corresponding author:

E-mail: lifeng2729@sxicc.ac.cn (F. Li); xiaofk@sxicc.ac.cn (F. Xiao).

Table of Contents

Fig. S1 TG-DTG curves of CAP-2 and CAP-X samples.

Fig. S2 SEM images of CAP-2, CAP-X, CA-2 and CA-X samples.

Fig. S3 Effect of reaction parameters on catalytic performance of the catalysts.

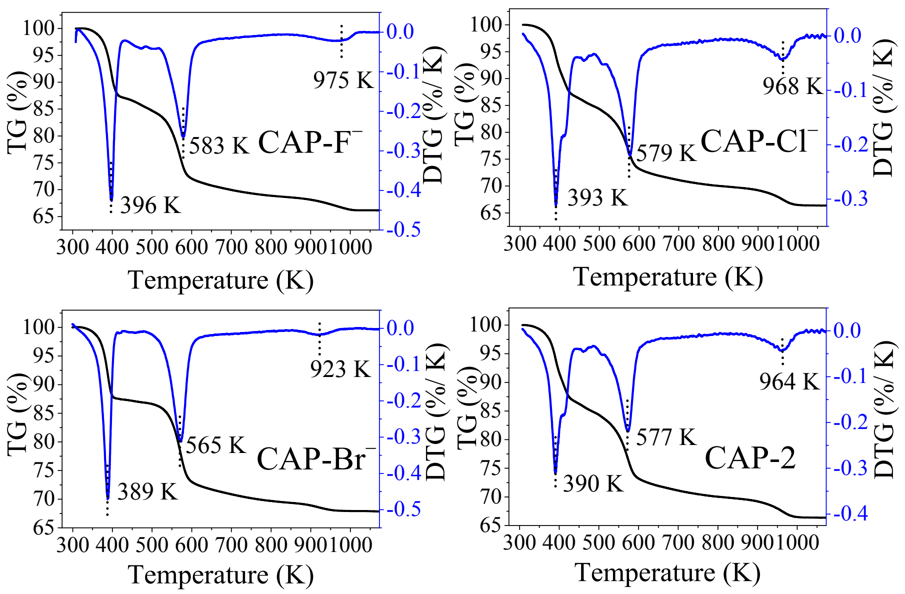

Fig. S1 TG-DTG curves of CAP-2 and CAP-X samples.

In order to demonstrates the effect of halogen anions on the thermal stability of the samples, the CAP-2 and CAP-X samples are analyzed by thermogravimetric technique and the TG-DTG curves of the samples are shown in Fig. S1. The decomposition of all samples can be classified into three stages.[1] A weight loss owing to the dehydration of the physically adsorbed water from the LDH is observed at 300–426 K. For the second stage in the range of 548–583 K, all samples transform to the corresponding oxides due to the dehydroxylation of the hydroxide layers. The third stage ranging from 871–983 K is corresponded to the further removal of the anions and the sintering of the Ca-Al oxides. From the results of DTG curves, the decomposition peaks of CAP-F^- and CAP-Cl^- samples shift toward higher temperature, suggesting the increase of the thermal stability. The introduction of F^- and Cl^- may increase the strength of hydrogen bonds and the electrostatic force between water molecules and interlayer anions which improve the thermal stability.[2] Particularly, it is interesting that the decomposition temperature of CAP-Br^- sample is

less than that of CAP-2 sample, which can be explained as that the larger ionic radii and lower anion affinity of Br^- may distort the LDH structure and thus lead to the decreased decomposition temperature.[3, 4] In a word, the thermal stability of the samples are in the order of CAP-F^- > CAP-Cl^- > CAP-2 > CAP-Br^-.

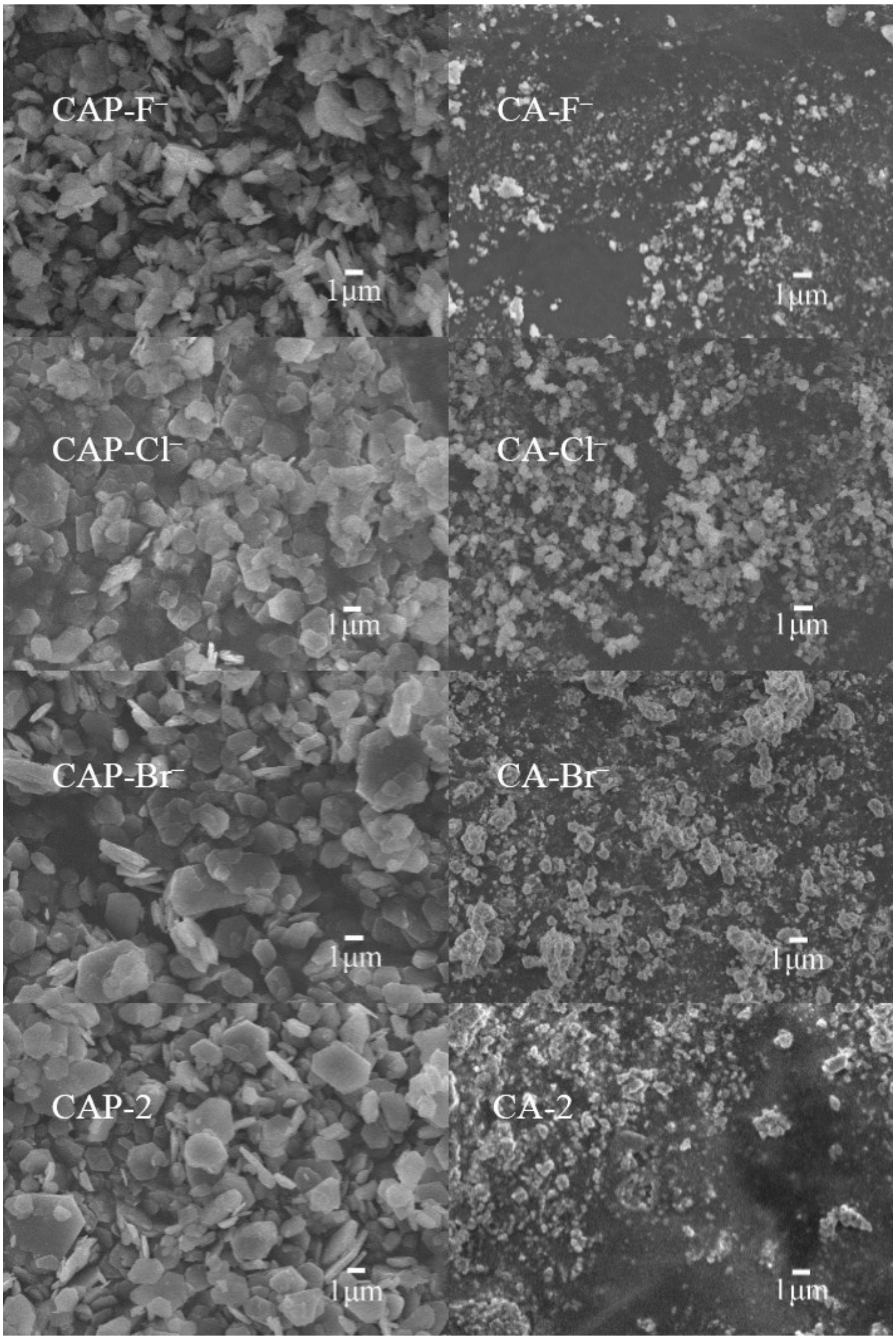

Fig. S2 SEM images of CAP-2, CAP-X, CA-2 and CA-X samples.

The scanning electron microscopy images of CAP-2 and CAP-X samples (Fig. S2) show typically hexagonal plate-shaped crystals which also suggest the formation of layered structure.[5] Compare to other samples, the shape of the CAP-F^- sample is more uniform, suggesting the better formation of the layered structure which is consistent with the result of XRD measurements (Fig. 1a). After calcination, the hexagonal plate-shape of the LDHs disappears and transforms into amorphous particles. The CA-2 and CA-X samples present the small and uniform particle size. Combined with the images of LDH precursors, the thinner and smaller plate of the CAP-F^- LDH precursor is more propitious to improve the metal dispersion, which leads to the formation of the smaller particles after calcination.

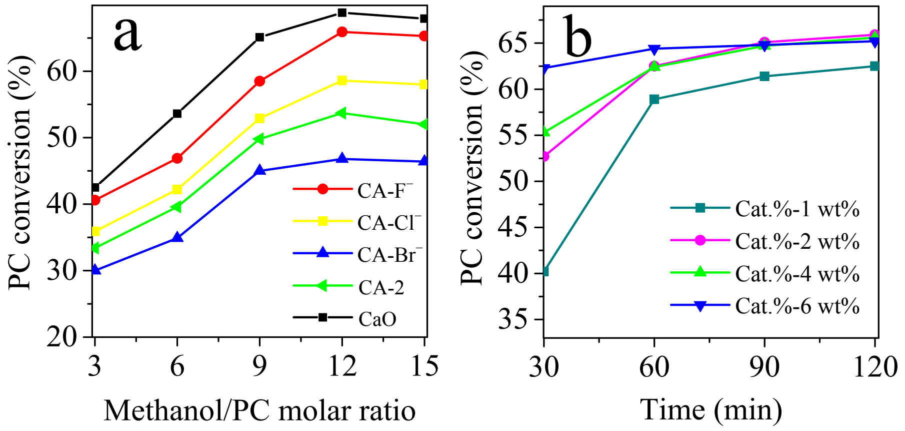

Fig. S3 Effect of reaction parameters on catalytic performance of the catalysts. Reaction conditions: (a) catalyst weight = 2 wt% of total reactants, T = 333 K, t = 2 h; (b) CA-F^- catalyst, n(methanol)/n(PC) = 12, T = 333 K.

Fig. S3a shows the effect of the methanol/PC molar ratio on the PC conversion. The higher methanol/PC molar ratio was in favor of the equilibrium of PC conversion owing to its reversible reaction. The PC conversion increased with the increased

methanol/PC molar ratio from 3 to 12, reached the highest value at 12, and then decreased a little when the molar ratio increased to 15, due to the insufficient PC which decreased the adsorption on the surface of the catalysts.[6] The effect of the catalyst weight on the catalytic performance was investigated and the results are shown in Fig. S3b. The PC conversion increased with the increased catalyst weight in a short time owing to the increased amounts of the active sites. As the catalyst weight exceeded 2 wt% and the reaction time exceeded 2 h, the PC conversion reached a plateau value and remained almost stable, suggesting that the reaction equilibrium had been reached.[7] Therefore, the optimized conditions of the transesterification were determined to be: methanol/PC molar ratio of 12, reaction time for 2 h and catalyst weight for 2 wt% of total reactants.

References

1 J. Kocík, M. Hájek and I. Troppová, *Fuel Process. Technol.*, 2015, **134**, 297–302.

2 E. Lima, J. Martinez-Ortiz Mde, R. I. Gutierrez Reyes and M. Vera, *Inorg. Chem.*, 2012, **51**, 7774–7781.

3 D. G. Costa, A. B. Rocha, W. F. Souza, S. S. X. Chiaro and A. A. Leitão, *Appl. Clay Sci.*, 2012, **56**, 16−22.

4 S. Miyata, *Clays Clay Miner.*, 1983, **31**, 305−311.

5 T. Baskaran, J. Christopher and A. Sakthivel, *RSC Adv.*, 2015, **5**, 98853–98875.

6 P. Kumar, V. C. Srivastava and I. M. Mishra, *Catal. Commun.*, 2015, **60**, 27–31.

7 M. K. Lam, K. T. Lee and A. R. Mohamed, *Appl. Catal. B*, 2009, **93**, 134–139.
